# Unique Three-Component Supramolecular Assembly for Highly Specific Detection of Zinc Ions

**DOI:** 10.3390/s25113470

**Published:** 2025-05-30

**Authors:** Xiaonan Geng, Lixin Zhang, Duan Xiong, Zhen Su, Qingqing Guan

**Affiliations:** Key Laboratory of Oil and Gas Fine Chemicals Ministry of Education, College of Chemical Engineering, Xinjiang University, Urumqi 830017, China

**Keywords:** three-component supramolecular, self-assembly, colorimetry, luminescence, platinum(II) complex, zinc(II)

## Abstract

The detection of zinc ions plays an essential role in protecting public health and maintaining ecological balance. However, traditional fluorescent probes for Zn^2+^ are limited in their specificity, especially under complex environments, due to their single-mode optical signal and inadequate recognization capacities. Herein we report a dual-mode supramolecular sensing system constructed from a unique three-component assembly involving a terpyridine platinum (II) complex, oxalate, and Zn^2+^, enabling highly specific detection performance for Zn^2+^. The supramolecular sensing system exhibits excellent selectivity among various interfering substances, accompanied by ultra-low detection limit (0.199 μM) and fast response (<3 s). The high recognization capacity comes from tri-component-based supramolecular assembly, while the dual-mode response arises from the generation of intermelcular Pt-Pt and π-π interactions, which yields absorption and emission originating from low-energy metal–metal-to-ligand charge transfer (MMLCT) transitions. This work marks a pioneering demonstration for highly specific detection of Zn^2+^ and inspires an alternative strategy for designing cation probes.

## 1. Introduction

As an indispensable trace element in the human body, zinc ions (Zn^2+^) play a vital role in numerous biological processes, including pathological mechanisms, brain function, gene transcription, immune response, DNA binding, apoptosis, mammalian reproduction, and muscle contraction [1,2,3,4,5,6]. Zn^2+^ is the second largest metal ion in the body after iron [7,8,9,10]. Abnormal Zn^2+^ concentrations can lead to a variety of health problems [11,12], including growth retardation [13], prostate cancer [14], diabetes [15], stroke, Alzheimer’s disease, and Parkinson’s disease [1,16]. In addition, Zn^2+^ is ubiquitous in daily life, ranking among the top ten common non-ferrous metals, with its consumption ranking third [17]. Spills caused by improper handling may trigger environmental contamination [18,19,20] and allow Zn^2+^ to enter the human body through the food chain [21,22]. Consequently, the achievement of highly specific detection of Zn^2+^ under complex environments is crucial for human health and environmental integrity.

According to previous reports, the quantification of Zn^2+^ ions has been accomplished through multiple analytical approaches such as atomic absorption spectrometry [23], inductively coupled plasma mass spectrometry [24], atomic emission spectrometry [25], and inductively coupled plasma electrical analysis methods [26]. However, these methods are not applicable for onsite rapid detection as there are obvious drawbacks like expensive instruments, experiential operation, and time-consuming pretreatment. Comparatively, the optical sensing method based on color or luminescence change has the advantages of simple operation, low cost, visualization, fast response, and high sensitivity [27,28,29,30]. So far, many probes have been developed for Zn^2+^ detection, such as Schiff base molecule [31], quinoline [32], dicyanisophorone [33], BODIPY [34], benzothiazole [35], amino-functionalized MOF [36,37,38], pyridinium fluorophore [39], and copper nanoclusters [40], however, their specificity under complex environments is inadequate. The inadequate specificity can be attributed to both the single-mode optical signal and inadequate recognition ability. On one hand, the single-mode optical signal is vulnerable to interferences with intrinsic color or automatic fluorescence. On another, the inadequate recognition ability leads false alarm from other co-existing competitive cations/anions. Therefore, it is highly desirable to develop an effective Zn^2+^ optical sensing system with both dual-mode optical signal and high recognition ability.

Recently, supramolecular platinum (II) complexes have been reported to be a promising colorimetric and luminescent dual-mode probe [41,42,43]. These complexes exhibit rich luminescence and colorimetry, arising from the formation of Pt-Pt interactions between non-radiative monomers during the supramolecular self-assembly process, leading low-energy metal–metal-to-ligand charge transfer (MMLCT) excited state [44]. Based on the unique optical properties and leveraging supramolecular self-assembly behavior, platinum(II) complexes have been successfully applied in the dual-mode detection of various ions, such as strontium(II) cations [45], mercury(II) cations [46], and perchloate anions [30,47]. However, these supramolecular sensing systems based on Pt-Pt interactions only involve two components and cannot completely eliminate interference from competing ions or molecules. The presence of certain metal ions can interfere with Pt-Pt interactions. For instance, anionic cyclometalated Pt(II) complexes exhibit cation-dependent luminescence phenomena [48], and the tetracyanoplatinate(II) (TCP) ion forms insoluble fluorescent compounds with many metal ions [49]. However, the detection of zinc ions faces significant interference from competing metal ions. How to eliminate interference from competing metal ions (particularly chromium) and endow the platinum (II) complex with high recognition capacity for Zn^2+^ detection requires in-depth investigation.

Three-component supramolecular self-assembly is an effective strategy for further enhancing recognition capabilities. This is because the additional components can generate extra interactions with the target ions, which not only strengthens the intensity of the recognition forces but also enriches the variety of recognition interactions [48]. Qing et al. ingeniously constructed a novel three-component self-assembly system based on triphenylamine-pyridine (TPA-Py), L-glutathione (L-GSH), and Ag^+^ successfully achieving high specificity recognition of Ag^+^ and L-GSH in water [50]. Glass et al. developed a three-component sensing system based on cucurbituril, pyridine derivatives, and fluorophores. They achieved high specificity recognition of amphiphilic glycosphingolipids through the hydrophobic interactions of cucurbituril with lipid moieties and the hydrophilic interactions of pyridine derivatives with glycan head groups [51]. The aforementioned studies provide valuable insights for enhancing the recognition capabilities of platinum (II) complex-based dual-mode probes.

Here, we have successfully prepared a unique three-component supramolecular sensing system based on terpyridine platinum (II) complex ([Pt(tpy)NCS]^+^), C_2_O_4_^2−^ and Zn^2+^, which show high recognition ability for Zn^2+^ and exhibit colorimetric and luminescent dual-mode optical signal, thus realizing highly specific detection of Zn^2+^. The successful construction of this three-component supramolecular system depends on first the coordination interaction between Zn^2+^ and C_2_O_4_^2−^ to form [Zn(C_2_O_4_)_3_]^4−^, which further triggers the ion-association and assembly reaction with [Pt(tpy)NCS]^+^ complex. The dual-mode response derives from the generation of intermolecular Pt-Pt and π-π interactions, which yields low-energy metal–metal-to-ligand charge transfer (MMLCT) absorption and emission. The supramolecular sensing system exhibits excellent selectivity among ~12 investigated interfering substances, accompanied by ultra-low detection limit (0.199 μM) and fast response (<3 s). This work represents an interesting demonstration for Zn^2+^ detection with high specificity and provides a new strategy for designing cation probes.

## 2. Materials and Methods

### 2.1. Materials and Reagents

All chemicals are analytical reagent grade and used as received. 1,5-cycloclooctadiene, potassium chloroplatinite (K_2_PtCl_4_), 2,2′:6′,2″-terpyridine, zinc chloride (ZnCl_2_), sodium oxalate (Na_2_C_2_O_4_), cadmium chloride (CdCl_2_), iron(III) chloride (FeCl_3_), aluminum chloride (AlCl_3_), cupric sulfate anhydrous (CuSO_4_), manganese chloride (MnCl_2_·4H_2_O), lithium chloride (LiCl_2_), calcium chloride anhydrous (CaCl_2_), nickel chloride hexahydrate (NiCl_2_·6H_2_O), cobalt(II) chloride (CoCl_2_), mercury(II) chloride (HgCl_2_), chromium(III) chloride (CrCl_3_), hydrochloric acid (HCl, 37 wt%), and sodium hydroxide (NaOH) purchased from Aladdin (Shanghai, China). Potassium thiocyanate (KSCN) purchased from Tianjin Fuchen Chemical Reagent Co., Ltd. (Tianjin, China). Acetic acid (AcOH), ethyl acetate (EtOAc), and dimethyl sulfoxide (DMSO) from Tianjin Xinbaite Chemical Co., Ltd. (Tianjin, China). All solutions are prepared using ultra-pure deionized water.

### 2.2. General Characterizations

^1^H NMR spectroscopy of the platinum (II) complex was performed on the Bruker 600 MHz spectrometer. Relative to tetramethylsilane (TMS) and D_2_O used as deuterated solvents, the chemical shift δ is given in parts per million (ppm). TMS served as the internal standard (δ = 0.00 ppm) for ^1^H NMR. The electrospray ionization mass spectra (ESI-TOF-MS) was acquired on an Agilent 6210 ESI/TOF mass spectrometer in positive ion mode using CH_3_CN. Elemental analysis was carried out on a Vario elementar CHNS analyzer. Power X-Ray Diffraction (PXRD) was recorded on a Rigaku SmartLab 9 kW diffractometer. The UV-visible spectrum was recorded on the SHIMADZU UV-2501PC UV-Visible spectrophotometer. Emission spectra were obtained on SHIMADZU RF-5301PC photoluminescence spectrometer. The optical micrographs were recorded on a Nikon Ci-E fluorescent upright microscope. Field emission scanning electron microscopy (FESEM) images were recorded on a Quanta 250 FEG. FT-IR spectra were measured with a PerkinElmer Spotlight 400 FT-IR microscope. The digital photos were taken with an iPhone 11.

### 2.3. Synthesis of [Pt(tpy)NCS]·SCN

[Pt(tpy)Cl]·Cl was prepared according to previously published procedures [52]. Complex [Pt(tpy)NCS]·SCN was obtained by adding excess KSCN to [Pt(tpy)Cl]·Cl, stirring for 30 min, draining, and filtering out precipitation, which became an orange dehydrated form after filtration, and dried in the oven for 10 h at 45 °C. Yield: 81%. Elemental analysis results (%): calculated for C_17_H_11_N_5_S_2_Pt: C 37.5; H 2.02; N 12.86. Found: C 36.83; H 1.96; N 12.62. ^1^H NMR (600 MHz, DMSO) δ (ppm): 8.63 (s, 1H), 8.61 (d, J = 10.7 Hz, 4H), 8.53 (td, J = 7.9, 1.5 Hz, 2H), 8.37 (d, J = 5.6 Hz, 2H), and 7.96 (ddd, J = 7.5, 5.6, 1.4 Hz, 2H) (Figure S1). ESI-MS: m/z calculated for [Pt(tpy)NCS]^+^: 486.4, found: 486.0.

### 2.4. Colorimetric and Luminescent Detection of Zinc(II)

The colorimetric and luminescent dual-mode sensing performance of Zn^2+^ in [Pt(tpy)NCS]^+^/C_2_O_4_^2−^ probe solution is tested by adding different concentrations of Zn^2+^ into the [Pt(tpy)NCS]^+^/C_2_O_4_^2−^ probe solution ([Pt(tpy)NCS]^+^: 0.8 mM, C_2_O_4_^2−^: 10 mM), and recording the absorption and luminescence spectra of the solution after 20 s. All luminescence spectra were determined at the excitation wavelength of 365 nm. In order to evaluate the selectivity of [Pt(tpy)NCS]^+^/C_2_O_4_^2−^ for Zn^2+^ detection, twelve common cations (Al^3+^, Ca^2+^, Cd^2+^, Co^2+^, Cr^3+^, Cu^2+^, Fe^3+^, Hg^2+^, Li^2+^, Mg^2+^, Mn^2+^, and Ni^2+^) were selected as interfering agents. The pH of [Pt(tpy)NCS]^+^/C_2_O_4_^2−^ solution was adjusted by HCl and NaOH in the range of 1–11 to detect Zn^2+^ at different pH conditions of probe solutions.

ESI-MS: m/z calculated for [Pt(tpy)NCS]_4_·[Zn(C_2_O_4_)_3_]: 486.0367, found: 486.0.

## 3. Results and Discussion

### 3.1. Three-Component Based Self-Assembly for Dual-Mode Detection of Zinc(II)

**Figure 1:**
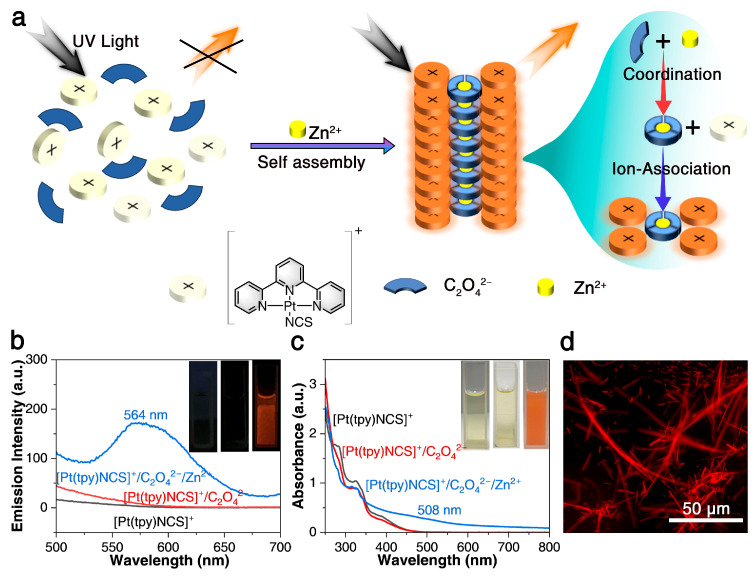
(**a**) Diagram of three-component supramolecular assembly for zinc ion dual-mode sensing. (**b**,**c**) Emission and absorption spectra of [Pt(tpy)NCS]^+^/C_2_O_4_^2−^ solution before and after the addition of Zn^2+^ (inset: images under 365 nm UV light and sunlight). (**d**) The optical microscopy image of [Pt(tpy)NCS]_4_·[Zn(C_2_O_4_)_3_] three-component supramolecular assembly aggregate in dark field.

In order to construct a three-component supramolecular dual-mode sensing system successfully, C_2_O_4_^2−^ was first introduced to coordinate Zn^2+^, because it has a high coordination constant (lgK_n_ = 8–9) with Zn^2+^ to form [Zn(C_2_O_4_)_3_]^4−^ coordination anions. In addition, the monocationic tripyridyl platinum (II) complex [Pt(tpy)NCS]^+^ was chosen because the monomer does not emit light in the aqueous medium due to the thermally accessible ^3^d-d excited state [42], and the π-conjugated tripyridyl ligands can produce rich photophysical properties through improved orbital interactions [53]. At the same time, according to previous studies of our research group, among the numerous tripyridyl platinum (II) complex, -NCS ligands can lead to tighter Pt-Pt interactions of crystals, lower band between HOCO and LUCO, and therefore more red-shift spectra are more likely to be achieved [45]. Therefore, when Zn^2+^, C_2_O_4_^2−^, and [Pt(tpy)NCS]^+^ encounter, Zn^2+^, C_2_O_4_^2−^ can be rapidly converted into [Zn(C_2_O_4_)_3_]^4−^ coordination anion in the initial stage, and then four [Pt(tpy)NCS]^+^ cations combine with [Zn(C_2_O_4_)_3_]^4−^ to form an ion pair. In the supramolecular self-assembly process, Pt-Pt and π-π interactions between non-radiating [Pt(tpy)NCS]^+^ are formed, resulting in significant color changes and turn-on luminescence, as shown in Figure 1a.

Subsequently, the colorimetric and luminescent dual-mode sensing properties of [Pt(tpy)NCS]^+^/C_2_O_4_^2−^/Zn^2+^ supramolecular system for Zn^2+^ are verified experimentally. First of all, it can be found that after the C_2_O_4_^2−^ is added to the [Pt(tpy)NCS]^+^ solution, the solution still maintains a non-luminous and light-yellow state, while the emission spectrum and absorption spectrum remain basically unchanged (Figure 1b,c). Subsequently, as shown in the insets in Figure 1b,c, with the addition of Zn^2+^, the originally non-luminous [Pt(tpy)NCS]^+^/C_2_O_4_^2−^ solution immediately becomes orange luminous under 365 nm UV light and changes from clear yellow to orange under sunlight, thus intuitively validating the dual-mode sensing with high-visibility color variation and luminous switch on. From the emission spectrum (Figure 1b), an obvious low-energy emission band appears at 564 nm after the addition of Zn^2+^, which can be attributed to the transition of MMLCT. At the same time, it can be seen from the UV-Vis absorption spectrum that the [Pt(tpy)NCS]^+^/C_2_O_4_^2−^ solution shows high-energy intraligand absorption at 335 nm and low-energy metal-ligand charge transfer (MLCT) absorption at 400 nm. After the addition of Zn^2+^, the absorption intensity at 335 nm decreased, while the new low energy absorption at 508 nm appeared, which was related to the MMLCT transition (Figure 1c). To further verify the detection process, we conducted FT-IR characterization of both the probe ([Pt(tpy)NCS]·SCN) and its aggregation products ([Pt(tpy)NCS]_4_·[Zn(C_2_O_4_)_3_]). As shown in Figure S2, the auxiliary ligand -NCS in the aggregates remained unchanged compared to the original probe, while the characteristic absorption peak of the counteranion -SCN in the initial probe structure was replaced by the newly introduced [Zn(C_2_O_4_)_3_]^4−^. Notably, the characteristic terpyridine (tpy) stretching vibration band at 3071 cm^−1^ remained consistent throughout the process. These spectroscopic observations conclusively demonstrate the Zn^2+^ -induced aggregation mechanism. We further characterized both [Pt(tpy)NCS]^+^ and [Pt(tpy)NCS]_4_·[Zn(C_2_O_4_)_3_] by PXRD (Figure S3), revealing significant shifts in the diffraction peaks, while confirming the crystal properties of the two materials and the transition of the crystal forms, proving the complete progress of the aggregation reaction. The morphologies of supramolecular self-assembled aggregates were observed by fluorescence microscopy. It was found that the aggregates showed orange glowing 1D aggregates in dark field (Figure 1d). The compositions of the formed 1D aggregates were further characterized using SEM and EDS mapping (Figure S4). These 1D aggregates exhibited diameters and lengths of ~5 µm and ~27 µm, respectively, with the characteristic Pt, C, N, S, O, and Zn elements dispersed uniformly. It was subsequently found that when the Zn^2+^ concentration increased from 0.2 mM and 3 mM to 15 mM, corresponding kinetic studies by monitoring the time-dependent absorbance intensity at 508 nm of the feature showed that the time to reach the reaction equilibrium was shortened from more than 20 s to 8 s, indicating that the probe had a super-fast response rate to Zn^2+^ (Figure S5).

### 3.2. Effect of pH on the Detection Performance

**Figure 2:**
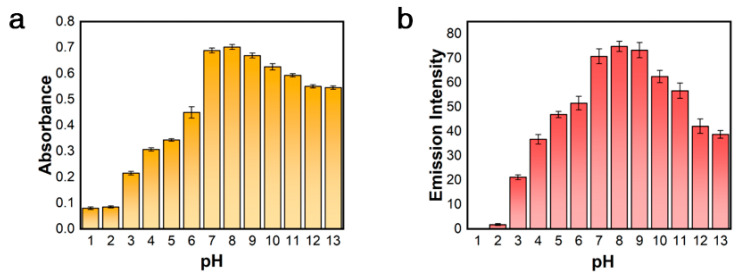
The effect of pH on (**a**) absorption intensity at 508 nm and (**b**) emission intensity at 564 nm of [Pt(tpy)NCS]^+^/C_2_O_4_^2−^/Zn^2+^ supramolecular system.

To assess the suitability of the [Pt(tpy)NCS]^+^/C_2_O_4_^2−^/Zn^2+^ supramolecular system, the effects of different pH values on the colorimetric and luminescent dual-mode detection of Zn^2+^ were investigated. As shown in Figure 2a,b, the supramolecular system exhibited significant pH dependence in the dual-mode detection of Zn^2+^. Regardless of whether the pH value is below 7 or above 10, both the absorption intensity at 508 nm and fluorescence intensity at 564 nm of supramolecular system also significantly decreased. In particular, colorimetric and fluorescence signals are almost undetectable at pH 1–2, which may be due to the protonation of C_2_O_4_^2−^ under strong acid conditions, making it difficult to coordinate with Zn^2+^.

However, the color and emission of [Pt(tpy)NCS]_4_·[Zn(C_2_O_4_)_3_] aggregates were relatively stable from pH 7–9. Therefore, we conclude that the correlation between pH and detection performance can be attributed to the influence of the acidity or alkalinity of the reaction medium on the formation of one-dimensional aggregates. The [Pt(tpy)NCS]^+^/C_2_O_4_^2−^ solution system under neutral or weakly alkaline conditions is more conducive to the formation of [Pt(tpy)NCS]_4_·[Zn(C_2_O_4_)_3_] aggregates, which is more favorable for Zn^2+^ sensing. Therefore, the detection process of Zn^2+^ by [Pt(tpy)NCS]^+^/C_2_O_4_^2−^/Zn^2+^ supramolecular system should be carried out under neutral or weakly alkaline conditions.

### 3.3. Sensitivity of [Pt(tpy)NCS]^+^/C₂O₄^2−^/Zn^2+^ Supramolecular System

**Figure 3:**
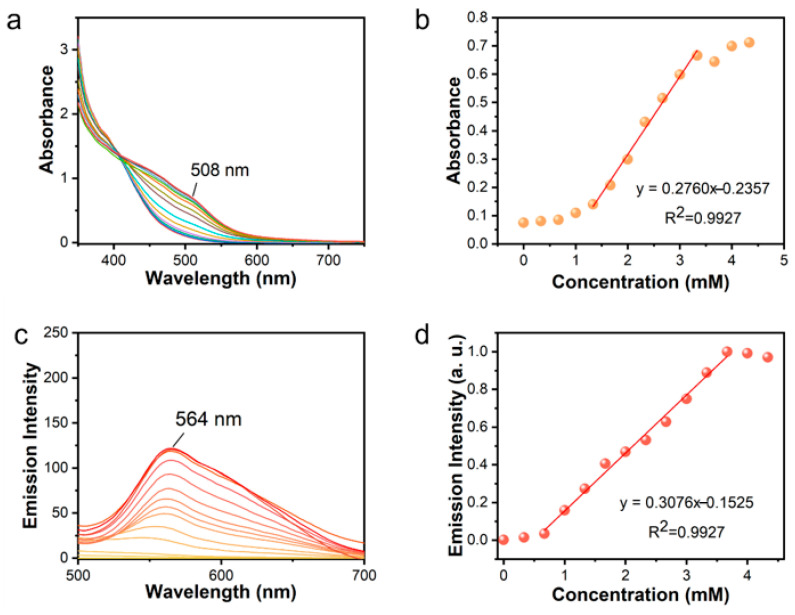
(**a**) Absorption spectrum variation of [Pt(tpy)NCS]^+^/C_2_O_4_^2−^ solution after adding Zn^2+^. (**b**) Absorption intensity at 508 nm as a function of Zn^2+^ concentration. (**c**) Fluorescence spectrum variation of [Pt(tpy)NCS]^+^/C_2_O_4_^2−^ solution after adding Zn^2+^. (**d**) Emission intensity at 564 nm as a function of Zn^2+^ concentration (different color lines in (**a**,**c**) represent different concentrations of Zn^2+^ added).

The Zn^2+^ sensitivity of the [Pt(tpy)NCS]^+^/C_2_O_4_^2^⁻/Zn^2+^ supramolecular system was evaluated in the concentration range of 0–4.29 mM via colorimetric and luminescent dual-mode response. As expected, the UV-Vis spectra revealed that the 508 nm absorption band exhibited progressive enhancement in the [Pt(tpy)NCS]^+^/C_2_O_4_^2−^ system upon increasing Zn^2+^ concentrations (0–4.29 mM, Figure 3a). It can be observed that the characteristic absorption band at 508 nm cannot be distinguished until the Zn^2+^ concentration increases to 1.32 mM. When the concentration of Zn^2+^ is in the range of 1.32–3.3 mM, the absorption band increases sharply, and when the concentration of Zn^2+^ is more than 3.3 mM, the growth rate of the absorption peak at 508 nm significantly slows down. In order to further study the increasing behavior of the absorption peak intensity in the range of 1.32–3.3 mM of Zn^2+^, the absorption intensity at 508 nm and Zn^2+^ concentration were linearly fitted (Figure 3b). Applying this linear range to limit of detection (LOD) (defined as LOD = 3σ/k, where σ is the standard deviation of the blank solution from ten independent measurements and k is the slope of the linear part of the fitted curve), the absorption LOD of Zn^2+^ is calculated to be 11.74 μM. At the same time, the luminescence sensitivity is shown in Figure 3c. Under the excitation of 365 nm, the characteristic emission peak of orange fluorescence at 564 nm increases with the increase of Zn^2+^ concentration, and the peak intensity at 564 nm has an obvious linear relationship in the range of 0.66–3.63 mM of Zn^2+^ concentration. According to the linear fitting slope of the luminescence intensity (Figure 3d), the luminescent LOD for Zn^2+^ at 0.199 μM can be obtained, which was much lower than the EPA level of Zn^2+^ in drinking water (∼76 μM). This is at a moderate level among existing probe detection levels (Table 1).

### 3.4. Selectivity of [Pt(tpy)NCS]^+^/C₂O₄^2−^/Zn^++^ Supramolecular System

**Figure 4:**
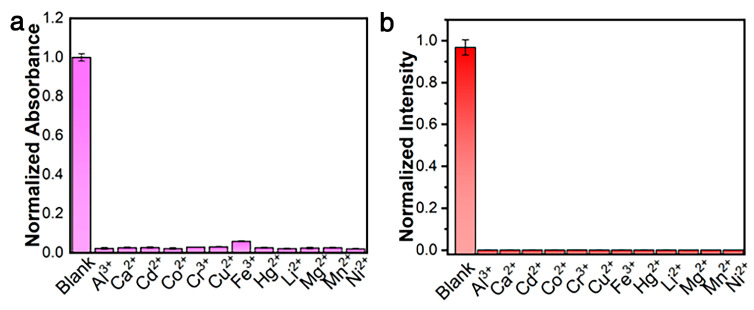
(**a**) Adsorption selectivity toward three-equivalent of common interfering ions. (**b**) Emission selectivity toward three-equivalent of common interfering ions.

To confirm the selectivity of the probe for Zn^2+^ detection, twelve common cations (Al^3+^, Ca^2+^, Cd^2+^, Co^2+^, Cr^3+^, Cu^2+^, Fe^3+^, Hg^2+^, Li^+^, Mg^2+^, Mn^2+^, and Ni^2+^) with concentrations three times higher were reacted with the [Pt(tpy)NCS]^+^/C_2_O_4_^2−^ and compared with Zn^2+^. The colorimetric and fluorescence responses of [Pt(tpy)NCS]^+^/C_2_O_4_^2−^ to various metal ions are shown in Figure 4a,b. By comparing the dual-mode signal intensity of other common metal ions with Zn^2+^, it was found that except Zn^2+^, other metal ions could not cause specific absorption to change at 508 nm and could not cause switching orange luminescence at 564 nm. The introduction of C_2_O_4_^2−^ enhances the recognition ability of Zn^2+^, and the detection method of colorimetric and luminescent dual channels reduces the possibility of false positive. The results show that the [Pt(tpy)NCS]^+^/C_2_O_4_^2−^/Zn^2+^ supramolecular system has good selectivity for the two-mode detection of Zn^2+^.

A great number of fluorescent probes for detection of Zn^2+^ display poor specificity and single signal changes, which reduce detection accuracy. In particular, the detection specificity is often interfered with by heavy and transition metal ions, especially Cd^2+^, because Cd^2+^ and Zn^2+^ show similar properties due to being located in the same group of the periodic table. Therefore, it is vital to develop highly specific probes to enhance accuracy of Zn^2+^ detection. As shown in Table 1, our probe can meet these requirements, exhibiting excellent specificity, colorimetric and luminescent dual-mode response, rapid response time, and high sensitivity.

### 3.5. Anti-Interference Ability of [Pt(tpy)NCS]^+^/C₂O₄^2−^/Zn^2+^ Supramolecular System

**Figure 5:**
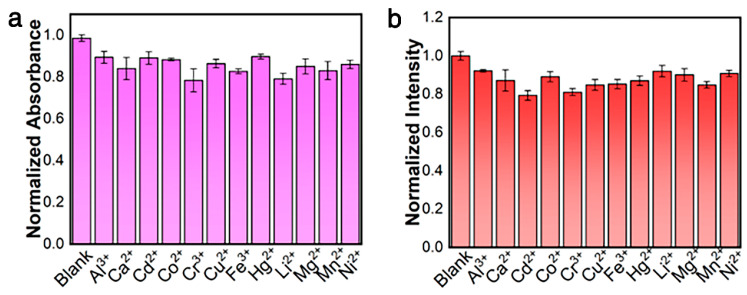
(**a**) Adsorption anti-interference ability toward three-equivalent of common interfering ions. (**b**) Emission anti-interference ability toward three-equivalent of common interfering ions.

The anti-interference ability of [Pt(tpy)NCS]^+^/C_2_O_4_^2−^/Zn^2+^ supramolecular system to Zn^2+^ detection was discussed. In the [Pt(tpy)NCS]^+^/C_2_O_4_^2−^ solution, changes in the intensity of absorption and emission were recorded when three times equivalent interfering metal ions coexisted with Zn^2+^, and the results are shown in Figure 5a,b. It can be observed that the presence of all interfering metal ions has no significant effect on the two-mode signal. The results show the excellent anti-interference performance of [Pt(tpy)NCS]^+^/C_2_O_4_^2−^/Zn^2+^ supramolecular system for Zn^2+^ detection.

## 4. Conclusions

In summary, we have developed a new unique three-component supramolecular sensing system based on terpyridine platinum (II) complex ([Pt(tpy)NCS]^+^), C_2_O_4_^2−^ and Zn^2+^ for specific recognition of Zn^2+^ in complex environments with colorimetric and luminescent dual-mode sensing signals. The recognition ability of Zn^2+^ in supramolecular system was enhanced by the introduction of C_2_O_4_^2−^, and the ternary supramolecular self-assembly of [Pt(tpy)NCS]^+^ was successfully induced by the formation of [Zn(C_2_O_4_)_3_]^4−^ coordination anions. Establishing Pt-Pt and π-π stacking interactions in supramolecular self-packed polymers makes the system change from light yellow to orange (508 nm) and turn on orange luminescence (564 nm), which can greatly resist environmental perturbation and improve detection accuracy. Moreover, the chemosensor possessed superior sensing performance with an ultra-low detection limit (0.199 μM), ultra-rapid response time (<3 s), remarkable specificity in identifying Zn^2+^, and anti-interference capability, facilitating its applications in complex environments. Together, this work provides an interesting demonstration of the highly specific detection of Zn^2+^ and a new strategy for the design of cation probes.

## Figures and Tables

**Table 1:** Comparison of detection performance between this probe and other probes for the detection of zinc ions.

Material	Specificity	Optical Signal	Response Time	LOD (μM)	Ref.
[Pt(tpy)NCS] ^+^/C_2_O_4_^2−^	High selectivity	Fluorescence colorimetric	3 s	0.199	Our work
Dicyanisophorone	Cd^2+^	Fluorescence colorimetric	15 s	0.21	[54]
Iminocoumarin derivatives	Cd^2+^, Fe^2+^, Co^2+^, Cu^2+^	Fluorescence turn-on	-	-	[55]
Spirobifluorene-based	Cu^2+^, Hg^2+^	Fluorescence enhancement	-	0.3	[56]
Dicyanoisoflurone derivative	High selectivity	Fluorescence colorimetric	30 s	0.0048	[33]
Quinoline-based	High selectivity	Fluorescence turn-on	2 s	0.025 ± 0.05	[57]
Pyridinium probe	Cd^2+^, Cu^2+^, Hg^2+^	Fluorescence enhancement	-	0.6	[39]
MOF	High selectivity	Fluorescence quenching	40 s	0.0314	[36]
Fluorescent probe TZn	High selectivity	Fluorescence colorimetric	40 s (50 μM)	0.31	[58]
Benzothiazole derivatives	Highselectivity	Fluorescence turn-on	10s	0.0236	[35]
Coumarin-based	High selectivity	Fluorescence enhancement	-	0.228	[59]
Quinoline-based	High selectivity	Fluorescence turn-on	-	0.063	[60]
(N, S-CD)-based	High selectivity	Fluorescence turn-on	40 min	0.005	[61]
Naphthofluorescein-based	High selectivity	Fluorescence enhancement	20 min	0.74	[62]
Quinoline chelator	Cd^2+^	Fluorescence enhancement	-	0.6	[63]
Schiff bases- based	Cd^2+^	Fluorescence enhancement	-	5.9	[64]
BODIPY-based	Cd^2+^, Co^2+^, Hg^2+^, Cu^2+^	Fluorescence enhancement	-	0.59	[65]

## Data Availability

The data are contained within the article or Supplementary Materials.

## Supplementary Materials

The following supporting information can be downloaded at: https://www.mdpi.com/article/10.3390/s25113470/s1, Figure S1: ^1^HNMR spectrum of [Pt(tpy)NCS]·SCN; Figure S2: FTIR spectra of [Pt(tpy)NCS]·SCN and [Pt(tpy)NCS]_4_·[Zn(C_2_O_4_)_3_]; Figure S3: PXRD of [Pt(tpy)NCS]·SCN and [Pt(tpy)NCS]_4_·[Zn(C_2_O_4_)_3_]; Figure S4: SEM and EDS image of the 1D aggregates; Figure S5: Effect of the Zn^2+^ concentration on the absorbance variation rate at 508 nm (Zn^2+^ concentration is 0.2 mM, 3 mM, and 15 mM).

## References

[1] Que E.L., Domaille D.W., Chang C.J. (2008). Metals in Neurobiology: Probing their Chemistry and Biology with Molecular Imaging. Chem. Rev..

[2] Frederickson C.J., Koh J.Y., Bush A.I. (2005). The Neurobiology of Zinc in Health and Disease. Nat. Rev. Neurosci..

[3] Zyba S.J., Shenvi S.V., Killilea D.W., Holland T.C., Kim E., Moy A., Sutherland B., Gildengorin V., Shigenaga M.K., King J.C. (2017). A Moderate Increase in Dietary Zinc Reduces DNA Strand Breaks in Leukocytes and Alters Plasma Proteins without Changing Plasma Zinc Concentrations. Am. J. Clin. Nutr..

[4] Hojyo S., Fukada T. (2016). Roles of Zinc Signaling in the Immune System. J. Immunol. Res..

[5] Sankova M., Nikolenko V., Oganesyan M., Vinnik Y., Gavryushova L., Redina S., Rizaeva N., Sankov A., Bulygin K., Vovkogon A. (2024). Zinc Pathogenic Importance in Correcting Immunity and Restoring Public Health in the POST-COVID Period: An Overview. Cytokine.

[6] Maret W. (2013). Zinc Biochemistry: From a Single Zinc Enzyme to a Key Element of Life. Adv. Nutr..

[7] Senthil Murugan A., Vidhyalakshmi N., Ramesh U., Annaraj J. (2017). A Schiff’s Base Receptor for Red Fluorescence Live Cell Imaging of Zn^2+^ Ions in Zebrafish Embryos and Naked Eye Detection of Ni^2+^ Ions for Bio-analytical Applications. J. Mater. Chem. B.

[8] Tayade K., Bondhopadhyay B., Keshav K., Sahoo S.K., Basu A., Singh J., Singh N., Nehete D.T., Kuwar A. (2016). A Novel Zinc(II) and Hydrogen Sulphate Selective Fluorescent “Turn-on” Chemosensor Based on Isonicotiamide: Inhibit Type’s Logic Gate and Application in Cancer Cell Imaging. Analyst.

[9] Chasapis C.T., Ntoupa P.-S.A., Spiliopoulou C.A., Stefanidou M.E. (2020). Recent Aspects of the Effects of Zinc on Human Health. Arch. Toxicol..

[10] Katimba H.A., Wang R.C., Cheng C.L., Zhang Y.C., Lu W.H., Ma Y. (2024). Zinc Absorption & Homeostasis in the Human Body: A General Overview. Food Rev. Int..

[11] Chen Y. (2023). The Relationship Between Zinc and Human Health and How to Supplement Zinc Scientifically. Theor. Nat. Sci..

[12] Singh P., Prasad S. (2023). A Review on Iron, Zinc and Calcium Biological Significance and Factors Affecting their Absorption and Bioavailability. J. Food Compos. Anal..

[13] Pratt E.P.S., Damon L.J., Anson K.J., Palmer A.E. (2021). Tools and Techniques for Illuminating the Cell Biology of Zinc. Biochim. Biophysica Acta (BBA)-Mol. Cell Res..

[14] Sauer A.K., Vela H., Vela G., Stark P., Barrera-Juarez E., Grabrucker A.M. (2020). Zinc Deficiency in Men Over 50 and its Implications in Prostate Disorders. Front. Oncol..

[15] Farooq D.M., Alamri A.F., Alwhahabi B.K., Metwally A.M., Kareem K.A. (2020). The Status of Zinc in Type 2 Diabetic Patients and its Association with Glycemic Control. J. Fam. Community Med..

[16] Viles J.H. (2012). Metal Ions and Amyloid Fiber Formation in Neurodegenerative Diseases. Copper, Zinc and Iron in Alzheimer’s, Parkinson’s and Prion Diseases. Coord. Chem. Rev..

[17] Abkhoshk E., Jorjani E., Al-Harahsheh M.S., Rashchi F., Naazeri M. (2014). Review of the Hydrometallurgical Processing of Non-sulfide Zinc Ores. Hydrometallurgy.

[18] Edokpayi J.N., Odiyo J.O., Popoola O.E., Msagati T.A.M. (2016). Assessment of Trace Metals Contamination of Surface Water and Sediment: A Case Study of Mvudi River, South Africa. Sustainability.

[19] Liu J., Peng A.G., Deng S., Liu M., Liu G.S., Li C. (2021). Distribution of Heavy Metals and Radionuclides in the Sediments and their Environmental Impacts in Nansha Sea Area, South China Sea. Mar. Pollut. Bull..

[20] Hama Aziz K.H., Mustafa F.S., Omer K.M., Hama S., Hamarawf R.F., Rahman K.O. (2023). Heavy Metal Pollution in the Aquatic Environment: Efficient and Low-cost Removal Approaches to Eliminate their Toxicity: A Review. RSC Adv..

[21] Mitra S., Chakraborty A.J., Tareq A.M., Emran T.B., Nainu F., Khusro A., Idris A.M., Khandaker M.U., Osman H., Alhumaydhi F.A. (2022). Impact of Heavy Metals on the Environment and Human Health: Novel Therapeutic Insights to Counter the Toxicity. J. King Saud Univ.-Sci..

[22] Adu J.T., Aneke F.I. (2025). Evaluation of Heavy Metal Contamination in Landfills from E-waste Disposal and its Potential as a Pollution Source for Surface Water Bodies. Results Eng..

[23] Li Q.M., Zhao X.H., Lv Q.Z., Liu G.G. (2007). The Determination of Zinc in Water by Flame Atomic Absorption Spectrometry After its Separation and Preconcentration by Malachite Green Loaded Microcrystalline Triphenylmethane. Sep. Purif. Technol..

[24] Płotka-Wasylka J., Frankowski M., Simeonov V., Polkowska Ż., Namieśnik J. (2018). Determination of Metals Content in Wine Samples by Inductively Coupled Plasma-Mass Spectrometry. Molecules.

[25] Hardaway C.J., Sneddon J., Sneddon E.J., Kiran B., Lambert B.J., McCray T.C., Bowser D.Q., Douvris C. (2016). Study of Selected Metal Concentrations in Sediments by Inductively Coupled Plasma-Optical Emission Spectrometry from a Metropolitan and More Pristine Bayou in Southwest Louisiana, United States. Microchem. J..

[26] Fakhari A.R., Shamsipur M., Ghanbari K. (2002). Zn(II)-selective Membrane lectrode Based on Tetra(2-aminophenyl) Porphyrin. Anal. Chim. Acta.

[27] Zhang H., Sun T., Ruan Q., Zhao J.L., Mu L., Zeng X., Jin Z.W., Su S.B., Luo Q.Y., Yan Y.Y. (2019). A Multifunctional Tripodal Fluorescent Probe for the Recognition of Cr^3+^, Al^3+^, Zn^2+^ and F^−^ with Controllable ESIPT Processes. Dyes Pigm..

[28] Xu Z.C., Yoon J., Spring D.R. (2010). Fluorescent Chemosensors for Zn^2+^. Chem. Soc. Rev..

[29] Li L.J., Wang J.H., Xu S.H., Li C.X., Dong B. (2022). Recent Progress in Fluorescent Probes for Metal Ion Detection. Front. Chem..

[30] Su Z., Li Y.S., Li J.G., Dou X.C. (2021). Ultrasensitive Luminescent Turn-on Detection of Perchlorate Particulates by Triggering Supramolecular Self-Assembly of Platinum(II) Complex in Hydrogel Matrix. Sens. Actuators B.

[31] Feng Q., Li Y.Y., Li K., Lu J.Y., Wang J.M., Fan P.Y., Li D., Wu D.M., Hou H.W. (2017). Fluorescent Chemosensor for Zinc Ion Detection with Significant Emission Color Change in Aqueous Solution Based on AIEgen. ChemistrySelect.

[32] Yun D., Chae J.B., So H., Lee H., Kim K.-T., Kim C. (2020). Sensing of Zinc Ions and Sulfide Using a Highly Practical and Water-soluble Fluorescent Sensor: Applications in Test Kits and Zebrafish. New J. Chem..

[33] Yan L.Q., Zhou C.P., Li J., Yang H., Wu X.Z., Li L. (2023). A near-infrared Fluorescent Probe Based on Dicyanisophorone for the Detection of Zinc Ions (Zn^2+^) in Water and Living Cells. J. Fluoresc..

[34] Xia S., Shen J.J., Wang J.B., Wang H.L., Fang M.X., Zhou H.W., Tanasova M. (2018). Ratiometric Fluorescent and Colorimetric BODIPY-based Sensor for Zinc Ions in Solution and Living Cells. Sens. Actuators B.

[35] Enbanathan S., Munusamy S., Jothi D., Manojkumar S., Manickam S., Iyer S.K. (2022). Zinc Ion Detection Using a Benzothiazole-based Highly Selective Fluorescence “Turn-on” Chemosensor and its Real-time Application. RSC Adv..

[36] Hameed Y.A.S., Alkhathami N., Snari R.M., Munshi A.M., Alaysuy O., Hadi M., Alsharif M.A., Khalil M.A., El-Metwaly N.M. (2025). Novel Amino-functionalized MOF-based Sensor for Zinc Ion Detection in Water and Blood Serum Samples. Spectrochim. Acta Part A.

[37] Liu X.L., Guo J.W., Wang Y.W., Wang A.Z., Yu X., Ding L.H. (2023). A Flexible Electrochemical Sensor for Paracetamol Based on Porous Honeycomb-like NiCo-MOF Nanosheets. Rare Met..

[38] Guo J.J., Zhao H.B., Yang Z.W., Wang L.W., Wang A.Z., Zhang J., Ding L.H., Wang L.F., Liu H., Yu X. (2024). Bimetallic Sulfides with Vacancy Modulation Exhibit Enhanced Electrochemical Performance. Adv. Funct. Mater..

[39] Thomas A., Nair A., Chakraborty S., Jayarajan R.O., Joseph J., Ajayaghosh A. (2024). A Pyridinium Fluorophore for the Detection of Zinc Ions Under Autophagy Conditions. J. Photochem. Photobiol. B.

[40] Lin L.Y., Hu Y.F., Zhang L.L., Huang Y., Zhao S.L. (2017). Photoluminescence Light-up Detection of Zinc Ion and Imaging in Living Cells Based on the Aggregation Induced Emission Enhancement of Glutathione-capped Copper Nanoclusters. Biosens. Bioelectron..

[41] Yang S.Y., Chen Y.Y., Kwok R.T.K., Lam J.W.Y., Tang B.Z. (2024). Platinum Complexes with Aggregation-Induced Emission. Chem. Soc. Rev..

[42] Su Z., Zhang L.X., Zhang H.Q., Li Y.S., Guan Q.Q. (2025). Biplane Ion-Pairing Induced Supramolecular Assembly for High-Performance Uranium Detection. Adv. Mater..

[43] Xie J.J., Xiao F.F., Liu C.G., Sun J., Yao J., Li Y.S. (2024). Effective Dual-mode Turn-on Sensing of Phosphates Enabled by the Twisted “Head-To-Head” Self-Assembly of a Platinum(II)-terpyridyl Complex with Close Pt–Pt Packing. J. Mater. Chem. C.

[44] Wong K.M.-C., Yam V.W.-W. (2011). Self-Assembly of Luminescent Alkynylplatinum(II) Terpyridyl Complexes: Modulation of Photophysical Properties through Aggregation Behavior. Acc. Chem. Res..

[45] Chen Y.Z., Pan D., Chen B., Wang G.X., Tung C.H., Wu L.Z. (2017). Synthesis, Characterization, and Selective Sr^2+^ Sensing Study of Copper(I)-Bridged Calix[4]Arene-Based Binuclear Alkynylplatinum(II) Complexes. Eur. J. Inorg. Chem..

[46] Cheng Y.K., Li L., Wei F.F., Wong K.M.-C. (2018). Alkynylplatinum(II) Terpyridine System Coupled with Rhodamine Derivative: Interplay of Aggregation, Deaggregation, and Ring-Opening Processes for Ratiometric Luminescence Sensing. Inorg. Chem..

[47] Su Z., Li Y.S., Li J.G., Li K., Dou X.C. (2022). Ultrasensitive Dual-mode Visualization of Perchlorate in Water, Soil and Air Boosted by Close and Stable Pt–Pt Packing Endowed Low-energy Absorption and Emission. J. Mater. Chem. A.

[48] Yoshida M., Kato M. (2020). Cation-controlled Luminescence Behavior of Anionic Cyclometalated Platinum(II) Complexes. Coord. Chem. Rev..

[49] Capelin B.C., Ingram G. (1970). Use of Tetracyanoplatinate (II) for the Luminescent Detection of Metalions. Talanta.

[50] Huang X.H., Li J.R., Tang H., Guo M., Wang X., Wang X.R., Wang X., Tang M.L., Zhang F.S., Zhang Y.H. (2023). Unique Three-component Co-assembly Among AIEgen, L-GSH, and Ag^+^ for the Formation of Helical Nanowires. Aggregate.

[51] Xu M., Kelley S.P., Glass T.E. (2018). A Multi-Component Sensor System for Detection of Amphiphilic Compounds. Angew. Chem. Int. Ed..

[52] Basolo F., Gray H.B., Pearson R.G. (1960). Mechanism of Substitution Reactions of Complex Ions. XVII.1 Rates of Reaction of Some Platinum(II) and Palladium(II) Complexes with Pyridine2. J. Am. Chem. Soc..

[53] Su Z., Li D.P., Zhang L.X., Tian S., Su Y.H., Hu X.Y., Xiong D., Guan Q.Q. (2024). Multiresponsive, Easy-Reversible, and Dual-visual Pt(II) Salt Nanostructures for Advanced Anti-counterfeiting Application. Nano Res..

[54] Yan L.Q., Lu D.Q., Yang H., Wu X.Z. (2023). A Dicyanisophorone-based Probe for Dual Sensing Zn^2+^ And Cd^2+^ by Near-infrared Fluorescence. Spectrochim. Acta Part A.

[55] Komatsu K., Urano Y., Kojima H., Nagano T. (2007). Development of an Iminocoumarin-based Zinc Sensor Suitable for Ratiometric Fluorescence Imaging of Neuronal Zinc. J. Am. Chem. Soc..

[56] Wan J., Zhang W., Guo H.D., Liang J.J., Huang D.Y., Xiao H.B. (2019). Two Spirobifluorene-based Fluorescent Probes with Aggregation-Induced Emission Properties: Synthesis and Application in the Detection of Zn^2+^ and Cell Imaging. J. Mater. Chem. C.

[57] Li W., Liu Z., Fang B., Jin M., Tian Y. (2020). Two-photon Fluorescent Zn^2+^ Probe for Ratiometric Imaging and Biosensing of Zn^2+^ in Living Cells and Larval Zebrafish. Biosens. Bioelectron..

[58] Zhang Y.B., Wang B.L., Rong X.Q., Liu J., Qiu X.Y., Sun L., Cheng Y.T. (2023). Development of a Novel Near-infrared Fluorescent Probe for Selective Detection of Zinc Ions in Environmental and Food Samples. Tetrahedron Lett..

[59] Yang W.S., Yang W., Ma Y.J., Yan L. (2025). A New Coumarin-based Fluorescent Chemosensor for Selection Detection of Zinc Ions in Aqueous Ethanol. Luminescence.

[60] Song H.H., Zhang Z. (2019). A Quinoline-based Ratiometric Fluorescent Probe for Discriminative Detection of Zn^2+^ and Cd^2+^ with Different Binding Modes, and its Zn^2+^ Complex for Relay Sensing of Pyrophosphate and Adenosine Triphosphate. Dyes Pigm..

[61] Chen S.Y., Li S.P., Liu X., Shi B.F., Huang Y.J., Zhao S.L. (2022). Nitrogen and Sulfur Co-doped Carbon Dot-based Ratiometric Fluorescent Probe for Zn^2+^ Sensing and Imaging in Living Cells. Microchim. Acta.

[62] Chen X.W., Xu J.J., Suo F.T., Yu C.M., Zhang D.T., Chen J., Wu Q., Jing S., Li L., Huang W. (2020). A Novel Naphthofluorescein-based Probe for Ultrasensitive Point-of-care Testing of Zinc(II) Ions and its Bioimaging in Living Cells and Zebrafishes. Spectrochim. Acta Part A.

[63] Jung J.M., Lee S.Y., Nam E., Lim M.H., Kim C. (2017). A Highly Selective Turn-on Chemosensor for Zn^2+^ in Aqueous Media and Living Cells. Sens. Actuators B.

[64] Mandal S., Sikdar Y., Maiti D.K., Sanyal R., Das D., Mukherjee A., Mandal S.K., Biswas J.K., Bauzá A., Frontera A. (2017). New Pyridoxal Based Chemosensor for Selective Detection of Zn^2+^: Application in Live Cell Imaging and Phosphatase Activity Response. J. Photochem. Photobiol. A.

[65] Lin J.R., Chu C.J., Venkatesan P., Wu S.P. (2015). Zinc(II) and Pyrophosphate Selective Fluorescence Probe and its Application to Living Cell Imaging. Sens. Actuators B.

