# Supplementary material for: Unique Three-Component Supramolecular Assembly for Highly Specific Detection of Zinc Ions
Source: Sensors (Basel). 2025 May 30;25(11):3470. doi: 10.3390/s25113470 (PMC12158297; doi:10.3390/s25113470)
Supplement: Supplementary file 1 [file sensors-25-03470-s001.zip › sensors-3554656-supplementary.pdf]

Supporting information for

Unique three-component supramolecular assembly for highly specific detection of zinc ions

Xiaonan Geng†, Lixin Zhang†, Duan Xiong, Zhen Su*, Qingqing Guan*

Key Laboratory of Oil and Gas Fine Chemicals Ministry of Education, College of Chemical Engineering, Xinjiang University, Urumqi 830017, China

†These authors contributed equally to this work

*E-mail addresses: zhensu@xju.edu.cn (Z. Su); guanqq@xju.edu.cn (Q. Q. Guan)

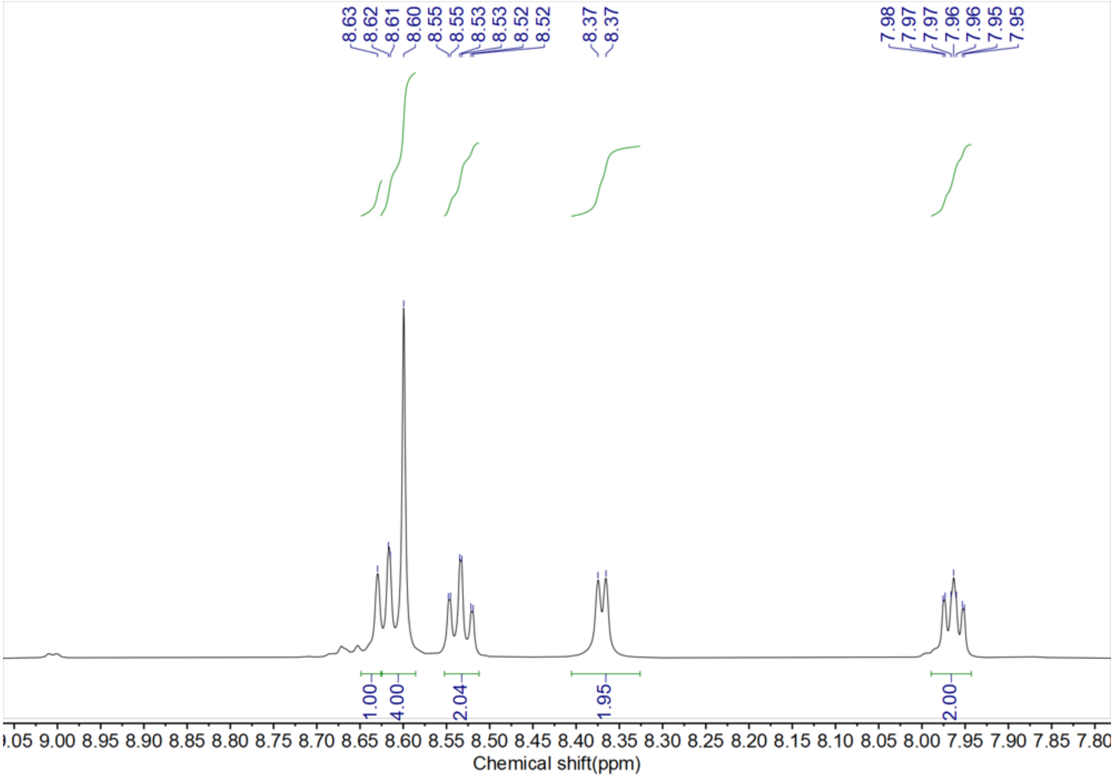

Figure S1. ^{1}HNMR spectrum of [Pt(tpy)NCS]·SCN.

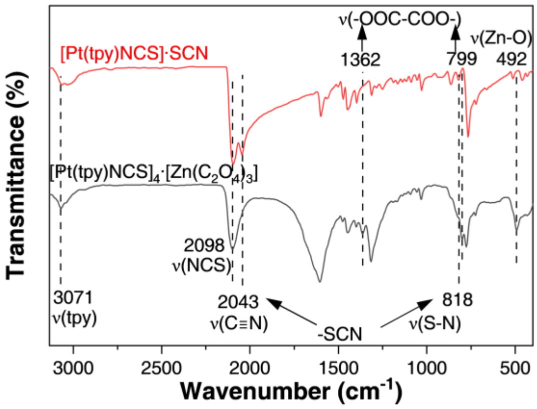

Figure S2. FTIR spectra of [Pt(tpy)NCS]·SCN and [Pt(tpy)NCS]$_4$·[Zn(C_2O_4)$_3$].

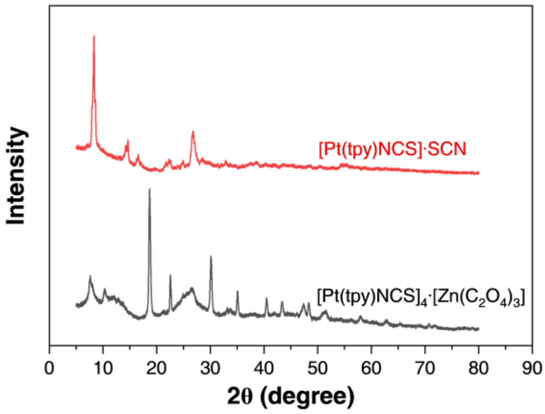

Figure S3. PXRD of [Pt(tpy)NCS]·SCN and [Pt(tpy)NCS]$_4$·[Zn(C_2O_4)$_3$].

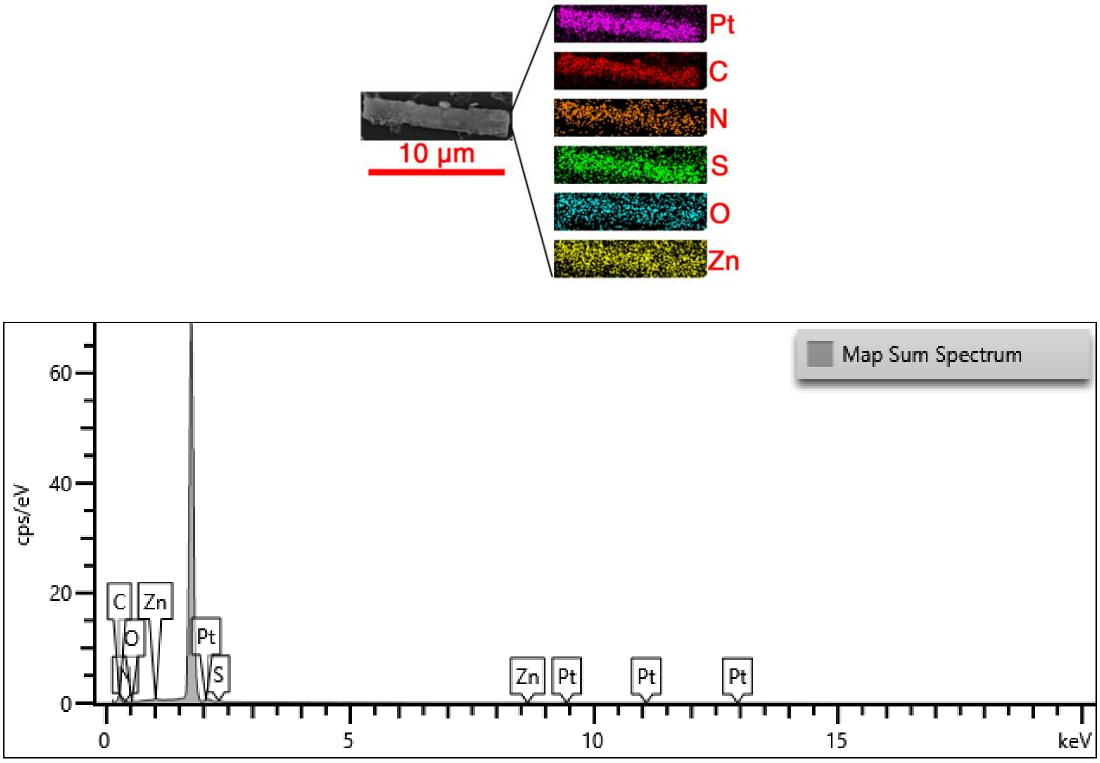

Figure S4. SEM and EDS image of the 1D aggregates.

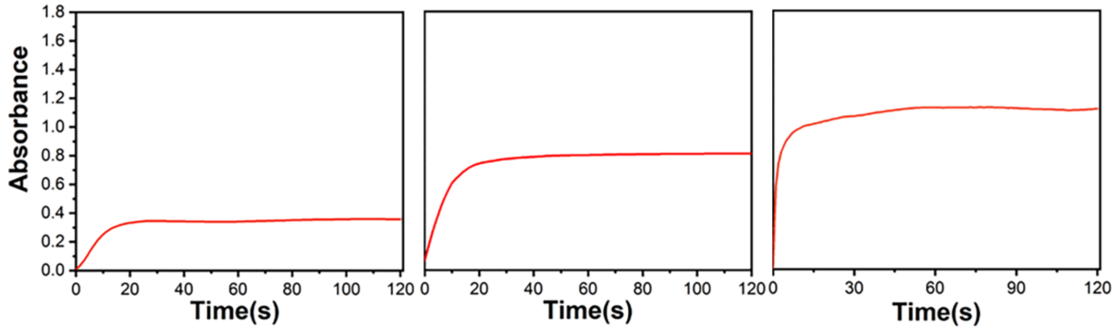

Figure S5. Effect of the Zn^{2+} concentration on the absorbance variation rate at 508 nm (Zn^{2+} concentration is 0.2 mM, 3 mM, 15 mM).
